# Effectiveness of a culturally tailored SMS alcohol intervention for same-sex attracted women: protocol for an RCT

**DOI:** 10.1186/s12905-019-0729-y

**Published:** 2019-02-06

**Authors:** Rachel Bush, Rhonda Brown, Ruth McNair, Liliana Orellana, Dan I. Lubman, Petra K. Staiger

**Affiliations:** 1 School of Psychology, Deakin University, 221 Burwood Highway, Burwood, Australia; 2 School of Nursing and Midwifery, Deakin University, 221 Burwood Highway, Burwood, Australia; 3 Department of General Practice, The University of Melbourne, 200 Berkeley Street, Carlton, Australia; 4 Biostatistics Unit, Deakin University, 221 Burwood Highway, Burwood, Australia; 5 Turning Point, Eastern Health and Eastern Health Clinical School, Monash University, 110 Church St, Fitzroy, Australia; 6 Centre for Drug Use, Addiction and Anti-Social Behaviour Research (CEDAAR), Deakin University, Geelong, Australia

**Keywords:** Alcohol, Intervention, Short message service (SMS), Same-sex attracted women, Lesbian, Bisexual, Women, Randomized controlled trial (RCT)

## Abstract

**Background:**

There is a large disparity between alcohol treatment access and prevalence of hazardous drinking among same-sex attracted women (SSAW). Yet, this population typically report low satisfaction with care and a reluctance to attend mainstream health services. Currently, there are few culturally tailored services for SSAW available despite evidence indicating that many feel uncomfortable in mainstream services. This paper describes the protocol of a randomised controlled trial aimed at examining the impact of a culturally sensitive four-week short message service (SMS) alcohol intervention on SSAW’s alcohol intake, wellbeing, and engagement with alcohol treatment.

**Methods:**

A randomised controlled trial comparing a culturally tailored SMS intervention (The Step One Program) with a generic ‘thank you’ message, and a nested qualitative study to further explore the intervention’s feasibility and acceptability. The Step One Program was co-designed using an Intervention Mapping framework and engaging potential consumers in the developmental process. Participants are block randomised (1:1 ratio) and followed up at the completion of the intervention and at 12 weeks post-intervention. The primary outcomes are alcohol reduction (as measured by the Alcohol Use Disorders Identification Test and self-reported alcohol intake), wellbeing (as measured by the Personal Wellbeing Index – Adult), and help-seeking (as measured by the number of alcohol services accessed and frequency of access). Upon completion of the 12-week post-intervention survey, participants in the intervention group were contacted via email regarding a phone interview on intervention acceptability.

**Discussion:**

This study may have important implications for clinical practice, improve healthcare access and equity for SSAW, and provide direction for future research in this field. The outcomes of the current study may stimulate the development of other culturally tailored health programs for SSAW. The results will inform whether individually tailoring the messages according to content and delivery frequency may be warranted to increase its acceptability.

**Trial registration:**

This trial was registered with the Australian New Zealand Clinical Trials Registry (trial ID: ACTRN12617000768392).

## Background

Same-sex attracted women (SSAW) typically report low utilisation of alcohol treatment options [1] despite some evidence of higher levels of alcohol consumption than heterosexual women [2–4]. Research also indicates that many SSAW are reluctant to seek help for alcohol-related problems from mainstream clinical services as they report low satisfaction with their care, and have difficulty finding sensitive and appropriate services [5–8]. Such findings highlight the need for culturally tailored services that meet their specific needs, and increase equity and access to appropriate alcohol treatment. However, to our knowledge, no research has been published which examines interventions specifically for SSAW to facilitate alcohol reduction.

It has been suggested that problematic drinking among SSAW is often associated with stressors specific to their sexual identity or exacerbated by their sexual identity [5] and these stressors are typically revealed during the process of reducing or abstaining from drinking. Yet many health practitioners receive little or no lesbian, gay, bisexual, and transgender (LGBT) cultural sensitivity training, or education about LGBT health issues [9, 10], which means these significant issues are often not adequately addressed or considered [11].

eHealth services are gaining popularity, particularly with women [12], and emerging evidence indicates short message service (SMS) has the potential to assist individuals with reducing their alcohol intake. A systematic review of SMS interventions to prevent alcohol and substance abuse has been conducted [13]. Among the seven studies reporting feasibility and acceptability outcomes, six demonstrated evidence of both and one reported low acceptability [13]. Importantly, six studies included in the review demonstrated a significant reduction in alcohol use [13]. For example, Suffoletto and colleagues [14] explored the effectiveness of delivering alcohol-related assessments plus feedback via SMS to outpatients from four hospital emergency departments in the United States of America. Participants were randomised into three groups: receiving the weekly SMS drinking-related assessments plus feedback (group one, *n* = 384), receiving weekly SMS drinking-related assessment without feedback (group two, *n* = 196), or usual care (group three, *n* = 185). Participants in group one reported significantly less binge drinking days and drinks per drinking day while participants in groups two and three reported a significant increase on both measures [14].

SMS studies to date have not specifically targeted SSAW or LGBT populations. Rather, they have typically used mainstream samples of young adults or students [15–17]. To the author’s knowledge, one SMS study has specifically targeted a minority population of socially disadvantaged men [18]. Nonetheless, based on the available evidence, SMS appears to be an appropriate method for delivering a brief alcohol intervention to SSAW. In addition to being accessible and wide reaching [19, 20], it overcomes many commonly reported barriers preventing SSAW from seeking alcohol support. For example, studies using Australian community-based samples of SSAW have found a shared concern of feeling discriminated against or experiencing heterosexist attitudes from the health practitioner, fear of perceived stigma relating to their sexual orientation or fears of being judged for their alcohol use, and a concern that their sexual orientation will be pathologised [1, 6]. As well as overcoming help-seeking barriers, SMS interventions are potentially a safe starting point for SSAW who are considering reducing their alcohol intake but do not yet feel confident seeking face-to-face support.

In order to address the service gap for SSAW, we developed the *Step One Program*, which is a culturally tailored SMS intervention for SSAW. Although the term ‘tailored’ often refers to customisation for individuals, we will be ‘culturally tailoring’ the message content which Pasick, D’Onofrio, and Otero-Sabogal [21] have defined as “the development of interventions, strategies, messages, and materials to conform with specific cultural characteristics” (p. 145). The program aim is to facilitate alcohol reduction, improve wellbeing, and increase help-seeking among SSAW. The current paper describes the protocol for a randomised controlled trial (RCT) to evaluate the effectiveness of the Step One Program for SSAW, and the feasibility and acceptability of conducting an SMS intervention for SSAW.

## Research objectives

### Hypotheses

Compared to participants who receive generic ‘thank you’ messages, participants in the Step One Program will report at the end of the intervention (4 weeks) and 12 weeks post-intervention:

1. Significantly lower alcohol intake as measured by the Alcohol Use Disorders Identification Test (AUDIT) and self-report of number of standard drinks consumed in the previous 30 days.
2. Significantly higher wellbeing as measured by the Personal Wellbeing Index – Adult (PWI-A).
3. Significantly higher service engagement as indicated by the number of services accessed and frequency of access.

## Methods

### Study design

A mixed methods approach was employed with a two-group, parallel, single-blind RCT, and a nested qualitative study to further explore the intervention’s feasibility and acceptability. The trial is registered with the Australian New Zealand Clinical Trials Registry (trial ID: ACTRN12617000768392). Ethics approval was obtained from the Deakin University Human Research Ethics Committee (reference number: 2017–077).

### Procedure

**Fig. 1:**
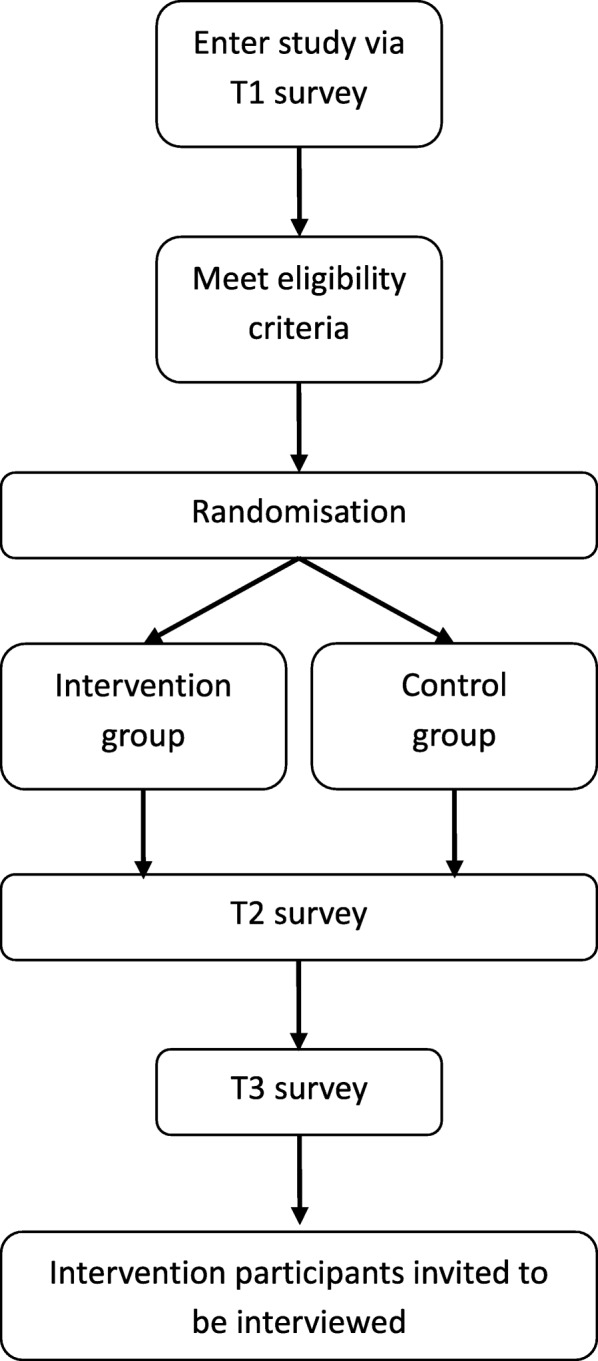
Procedure flowchart

The study procedure described in the following sections is presented in Fig. 1.

#### Participant recruitment

Recruitment commenced April 24, 2017 and data collection is ongoing. Participants are recruited from four sources:

1. General practice clinics across Australia that are known to have a high SSAW case-load;
2. Nation- and State-wide SSAW community and social networks through email, websites, and social media. General women’s health groups are also contacted to reach SSAW not active in the LGBT community;
3. Participants from the Rainbow Women’s Help-Seeking study (which examined professional and social help-seeking by SSAW) [7], and the ALICE study (which investigated socio-cultural factors which influenced alcohol use, sexual orientation, mental health, and health service use among SSAW) [1], with permission from the University of Melbourne;
4. Public common areas, such as restrooms and community noticeboards.

Individuals enrol in the study by completing the online baseline survey using a link included with all study advertisements. The home page of the survey provides detailed information about the study, what participation involves, that participants can withdraw at any time and if so, they will be requested (but not obligated) to complete a survey containing the primary outcome measures, that it is anonymous, and that all information collected is confidential. It is a requirement to indicate consent before proceeding to the survey. To acknowledge the time taken to participate, participants who complete the final follow-up survey will go into a draw to win one of two $50 retail vouchers.

#### Eligibility criteria

To be eligible, participants need to:

1. Identify as a same-sex attracted woman. This includes transgender women, transgender men, and gender diverse individuals. Transgender men were eligible as these individuals were likely involved in lesbian, bisexual, and queer women’s communities prior to transitioning to male; and if the individual has not undergone gender reassignment surgery, certain female health issues will still affect them.
2. Be aged 18 years or older.
3. Score eight or above on the AUDIT.
4. Own a mobile phone with SMS capabilities and have access to the internet.
5. Respond to both the welcoming email and the test SMS message received after enrolling.

#### Data collection

##### Baseline (T1)

Baseline data are collected using an online survey using *Qualtrics*. The survey primarily measures alcohol use, wellbeing, and current help-seeking. For a list of measures, please refer to Table 1. Participants are asked to provide their primary email address and mobile telephone number to send two follow-up surveys and to deliver the SMS intervention.

**Table 1:**
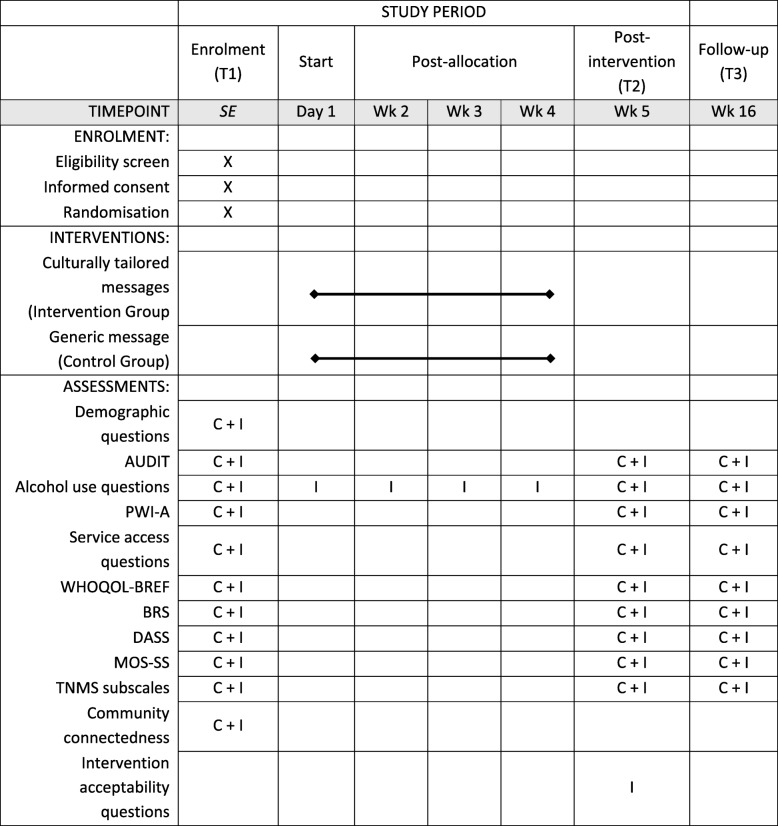
SPIRIT Flow Diagram

C = control group; I = intervention group; SE = study entry; AUDIT = the Alcohol Use Disorders Identification Test; PWI-A = the Personal Wellbeing Index – Adult; WHOQOL-BREF = the WHO Quality of Life-BREF; BRS = the Brief Resilience Scale; DASS = the Depression Anxiety Stress Scales; MOS-SS = the Medical Outcomes Study Social Support Survey; TNMS = the Treatment Needs and Motivation Scale

##### Post-intervention (T2)

Following the completion of the four-week intervention, a post-intervention online survey is emailed to participants. Participants in the intervention group are asked to respond to an additional set of questions regarding intervention acceptability. See Table 1 for a list of measures used in the T2 survey.

##### Follow-up (T3)

At 12 weeks post-intervention, all participants will complete a follow-up survey. See Table 1 for a list of measures used in the T3 survey.

#### Random allocation

Individuals are screened for eligibility. The first two eligibility criteria are determined in the baseline survey. If an individual responds that they identify as a man or they are aged under 18 years, they will be automatically directed to the end of the survey. The AUDIT score is calculated by RBu once the baseline survey is received. If the individual scores eight or above, they will then receive a welcome email and test SMS message. Once the receipt of both has been confirmed, participants are randomly allocated into the intervention group or the control group using a computer generated block randomisation at a 1:1 ratio with 10 allocations per block to ensure equal numbers in each group if the recruitment goal is not achieved. The sequence of condition allocations are placed in opaque envelopes with participant identification numbers on the front. Once a participant’s eligibility is determined, an envelope is opened by RBu and the participant is allocated to the experimental condition inside the envelope. As participants are blinded, to reduce bias they will not be informed of the number or frequency of messages in the intervention as this information will reveal which group participants have been randomly allocated to.

#### Intervention group

The intervention consists of automated culturally tailored supportive SMS messages which are delivered through *MessageMedia*, an Australian SMS platform. It is designed to begin on a Monday and end on a Sunday. Messages are delivered daily for four weeks with two messages on Thursdays, Fridays and Saturdays, as people typically drink alcohol on these days (40 messages in total). Messages are sent at varying times between 3:00 p.m. and 4:30 p.m., and on days with two messages, the second is delivered between 6:00 p.m. and 8:00 p.m. On Sundays, the message asks participants to reply via SMS with the number of standard drinks they have consumed in the past seven days. In the welcome email, all intervention participants receive a standard drinks chart and an author-developed list of LGBT specific or friendly alcohol and mental health services.

##### Intervention development

The intervention was co-designed with potential consumers and utilised an Intervention Mapping framework [22, 23] which provides a best practice process to intervention development with a strong focus of engaging potential consumers in the developmental process. This consisted of six steps to develop an evidence-based intervention with each step being cumulative so that the result of each step guided the next (Bush, R., Brown, R., McNair, R., Lubman, D. I., & Staiger, P. K.: Development of a tailored SMS alcohol intervention for same-sex attracted women using an intervention mapping framework, in preparation).

The first step involved a needs assessment to identify the gap in culturally tailored or appropriate alcohol treatment services available to SSAW. This included a comprehensive literature review and running focus groups with SSAW to explore whether they wanted a culturally tailored service and what they believed should be included. Step two involved developing a table of performance objectives (see Table 2) in which each cell of the table outlined what participants would need to learn or change in order to reduce their alcohol intake, improve their wellbeing, and increase their access to alcohol support services. The selection of these three primary outcomes was influenced by the literature and focus group discussions. Alcohol reduction was selected because research has consistently demonstrated a greater risk for hazardous alcohol use among SSAW compared to heterosexual women [3, 4, 24]. Wellbeing was selected as SSAW who drink hazardously also tend to have a lower level of general wellbeing as they commonly report experiences of sexual and physical abuse [25, 26], low social support [27, 28], and less access to housing, employment and healthcare [29–31]. Finally, help-seeking was selected as SSAW are typically reluctant to seek professional help due to a lack of services which are responsive to their unique needs [5, 8].

**Table 2:** Table of Performance Objectives

Performance Objectives	Targets of Change			
	Resilience	Mental Health	Social Support	Motivation to Change
Reduce alcohol intake	Able to cope with general and sexual orientation related stress and adversity in a healthy way rather than using alcohol as a coping mechanism.	Able to use healthier coping strategies to deal with psychological distress related to sexual orientation rather than using alcohol to self-medicate.	Make social connections where alcohol is not the focus of social gatherings.	Understand what can be gained by reducing alcohol intake, feels confident about the ability to successfully reduce intake, and makes it a priority to achieve this.
Improve wellbeing	Able to approach challenges with confidence, realistic optimism, and a sense of control.	Is aware of own potential and limits, and can cope with stress and challenges.	Increase time with positive social network who evoke self-confidence and optimism.	Understand what can be gained by being healthier, feels confident about ability to successfully make these changes, and will prioritise looking after health and well-being.
Increase help-seeking	Understand that by receiving positive support and help, aspects of resilience such as self-efficacy, hope, and coping, will be strengthened.	Is aware of general and SSAW sensitive mental health services and understand what can be gained by seeking support.	Is aware that informal supports (e.g. peers and support groups) can be positive sources of help and support but also understand that formal sources are important for significant emotional and mental health issues.	Is aware of the services that are available to SSAW, feels confident in ability to engage with the services, and will make it a priority to contact services for help and support.

Four mediating variables were included in the table of performance objectives (see Table 2). These were identified during the needs assessment and were deemed important for successful behaviour change among SSAW. Improving resilience was the first mediator identified and was selected because the main elements of resiliency, such as self-efficacy, hope, and coping [32], have the potential to help individuals cope with stress and adversity in a healthy way [33]. The second mediator, mental health, was deemed to be an influential factor in SSAW’s ability to improve the primary outcomes as they have been found to experience higher levels of depression and anxiety compared to heterosexual women [34], and SSAW who have poor mental health have been found to drink at hazardous levels [24, 35]. Facilitating social support was chosen as the third mediator because individuals who lack social support are more vulnerable to poor health outcomes [36]. Lastly, enhancing motivation to change was deemed an important factor as it can influence an individual’s desire to comply with and finish a treatment program [37].

Step three in the Intervention Mapping framework involved selecting behaviour change techniques which were operationalised in step four. The selection of behaviour change techniques was guided using three theoretical frameworks: the Information-Motivation-Behavioural Skills model [38], the Health Belief Model [39, 40], and the Theory of Planned Behaviour [41]. SSAW were consulted during step four which involved developing the SMS statements in order to receive their input on the messages to ensure the language and content was appropriate and perceived to be helpful. Table 3 displays some example SMS messages that were delivered to the intervention group. The current paper describes the process for implementing steps five and six which pertain to the delivery and evaluation of the intervention.

**Table 3:** Example SMS Messages Delivered to the Intervention Group

Target	Message Statements
Performance objective: Reduce alcohol intake	
Resilience	• Identify and write down triggers that make you want to drink. These may include social (ie discrimination, others drinking) or health (ie distress) factors.
Mental health	• It’s common to use alcohol to cope with distress related to discrimination. Write down some healthier ways to cope with distress, ie deep breathing, exercise.
Social support	• Does your partner/housemate drink? Try to reduce your drinking together. You can support each other and both stick to your goals when at a bar or pub.
Motivation to change	• What is your alcohol reduction goal? When and how will you start? Eg, Not to exceed 2 drinks when I’m out, starting on Saturday night.
Performance objective: Improve well-being	
Resilience	• Does visiting a new doctor make you feel distressed? Write down ways you can confidently approach this. Also try visiting doclist.com.au for a list of services.
Mental health	• LBQ+ women face everyday stress plus sexual orientation related stress and sexism. If you’re having a hard day, know your limits and give yourself a break.
Social support	• If you encounter discrimination, homophobia, abuse, try talking to someone who makes you feel good about yourself, ie your partner, a family member, or friend.
Motivation to change	• Health includes physical, mental and social aspects. Think of how/when you can improve these areas ie go walking with a friend on Saturdays.
Performance objective: Increase help-seeking	
Resilience	• Seeking support can help you identify or relearn healthy coping strategies. Visit or call QLife for support and referrals: qlife.org.au/support/ or 1,800,184,527
Mental health	• If you are feeling stressed/anxious/down etc. set aside a time in your calendar to make an appointment with someone who can help.
Social support	• Connecting with like-minded people is important. Support groups are a great addition to formal support as you will meet others who get what you’re experiencing.
Motivation to change	• Are bad past experiences stopping you from seeking support? Refer to the list of services that was emailed to you for LBQ+ women appropriate services.

#### Comparator group

Consistent with other trials that have delivered SMS alcohol interventions (e.g. [15, 42]), participants in the control group receive a generic weekly message: “Thank you for participating in this study. For LGBT specific information on drug/alcohol use, mental health and sexual health, visit http://touchbase.org.au”. These participants do not receive a standard drinks chart or a list of support services. At the end of the study, after completing the 12-week follow-up survey, participants receive the list of support services and are offered the chance to receive the intervention messages.

### Measures

#### Demographic information

The baseline survey includes: standard demographic questions, such as age, residential location and education; questions related to sexuality ask participants about their sexual identity, behaviour and attraction; questions related to gender identity asking whether they identify as female, transgender female, transgender male, non-binary, or another identity not listed; and questions related to relationship status, whether participants are currently in a relationship, with how many people, and the gender of their partner/s.

#### Alcohol use and severity

Severity of alcohol use is measured using a modified version of the AUDIT, a screening tool developed by the World Health Organisation [43]. The instrument includes ten questions answered on Likert scales assessing three domains: hazardous alcohol use, dependence symptoms, and harmful alcohol use. Questions three to 10 were changed to ask participants about drinking outcomes in the past four weeks at T2 and past 12 weeks at T3 rather than the past year to avoid collecting overlapping data. A score of 0–7 indicates ‘low-risk’ alcohol use; a score of 8–15 indicates a ‘hazardous level’ of alcohol use; a score of 16–19 indicates a ‘harmful level’ of alcohol use; and a score of 20 or more indicates ‘high-risk’ alcohol use. This scale has been validated and successfully used in different populations [43, 44].

Alcohol use is measured by asking participants to report the number of standard drinks they consumed in the previous 30 days. A basic standard drinks chart is included to assist with calculations.

#### Wellbeing

The PWI-A [45] is used to assess general wellbeing. This 7-item scale measures seven domains: standard of living, personal health, achieving in life, personal relationships, personal safety, community-connectedness, and future security. A supplementary item asks about satisfaction with life as a whole. Items are answered on a 10-point Likert scale ranging from 0 (no satisfaction at all) to 10 (completely satisfied). Australian and international research has demonstrated good reliability of the PWI-A [46].

The WHO Quality of Life-BREF (WHOQOL-BREF) [47] is used as it is a comprehensive measure of wellbeing and quality of life. This instrument contains 26 questions measuring four domains: physical health, psychological, social relationships, and environment. In Australia, the physical health, psychological, and environment domains have been found to have acceptable internal reliability, and marginal internal reliability was found for the social relationships domain [48].

#### Service access and engagement

Participants are presented with a list of alcohol reduction services and treatments. These are: a general practitioner (GP), another doctor (e.g. specialist doctor), a nurse from your general practice, another nurse, social worker, counsellor/psychologist/psychiatrist you attended in person, general counselling telephone helpline (e.g. Lifeline [a free 24/7 telephone crisis hotline]), Counselling Online (a free 24/7 online text-based support for individuals affected by alcohol and other drugs), other drug or alcohol telephone helpline, drug or alcohol service you attended in person, drug or alcohol self-help group (e.g. AA), hospital emergency department, police, naltrexone, acamprosate, disulfiram, and other. At T1 they are asked to indicate which ones they are currently accessing. At T2 and T3, the same list is presented and participants indicate how frequently they accessed each service in the past four weeks at T2 and past 12 weeks at T3 (did not use, 1–2 times, 3–5 times, 6–9 times, 10+ times). Participants can list additional services not included in the survey and are asked to indicate whether any of the services are LGBT or SSAW-specific.

#### Resilience

Resilience is measured using the Brief Resilience Scale (BRS) [49], a 6-item scale measuring ability to recover from stress. Each item is answered on a 5-point Likert scale ranging from 1 (strongly disagree) to 5 (strongly agree). Strong internal reliability has been demonstrated in a sample of women with a mean age of 47.3 years [49].

#### Depression, anxiety, and stress

The Depression Anxiety Stress Scales (DASS) is a 42-item scale with three subscales: depression, anxiety, and stress [50]. Items reflect a negative emotional indicator and are answered on a 4-point Likert scale ranging from 0 (did not apply to me at all) to 3 (applied to me very much, or most of the time). Scores of 10 or more on the depression subscale, 8 or more on the anxiety subscale, and 15 or more on the stress subscale indicate higher than ‘normal’ experiences of each subscale. In a non-clinical general sample of adults, strong internal reliability has been found for the subscales and total score [51].

#### Social support

Social support is measured using the Medical Outcomes Study Social Support Survey (MOS-SS), a 19-item scale [52]. The items are answered on a 5-point Likert scale ranging from 1 (none of the time) to 5 (all of the time) and ask questions related to emotional/informational support, tangible support, affectionate support, and positive social interaction. Research has demonstrated sound psychometric properties for this survey [52, 53].

#### Motivation to change

Motivation to change is measured using the Treatment Needs and Motivation Scale (TNMS) [54]. This scale consists of 36 items and five subscales. The current study administered questions from the Problem Recognition, the Desire for Help, and the Treatment Readiness subscales which is a total of 23 items answered on a 5-point Likert scale ranging from 1 (strongly disagree) to 5 (strongly agree). Items from the Pressures for Treatment and Treatment Needs subscales are not included as they were not deemed to be relevant to the current study. The scale items were reworded as the phrase “drug use” was replaced with “alcohol use”. Internal reliability has been demonstrated for the three subscales [55].

#### Community connectedness

Community connectedness is measured using the Connectedness to the LGBT Community Scale [56]. A modified version that was used in the Rainbow Women’s Help-Seeking Study [7] is also included to measure connectedness to the mainstream community. The original scale was modified to remove references to the LGBTI community in New York and instead ask about the LGBT community in general. Both scales contain 7 items answered on a 4-point Likert scale ranging from 1 (strongly disagree) to 4 (strongly agree). Good internal reliability has been demonstrated for the LGBT and mainstream versions in an Australian sample of sexual minority women [7].

#### Intervention acceptability

At T2, intervention group participants are asked questions that were adapted from two separate studies [57, 58]. They are asked: how often they read the SMS messages (always, often, sometimes, rarely); if the times they received the SMS messages were appropriate (yes, no); how satisfied they were with the frequency of the SMS messages (very satisfied to very dissatisfied); how frequently they would have preferred to receive messages (more than twice daily, twice daily, once daily, at least once per week, never); how helpful they found the SMS messages using a 5-point Likert scale ranging from 1 (extremely unhelpful) to 5 (extremely helpful); if they would recommend SMS messages as an intervention for other SSAW (most certainly, probably, not sure, certainly not); and the importance of culturally tailored message content, measured using an author developed question on a 5-point Likert scale ranging from 1 (unimportant) to 5 (extremely important).

### Data analysis

All data will be de-identified and coded to ensure participant anonymity and it will stored securely on a password protected computer. Participant names, corresponding identification numbers, and contact information will be kept in a separate password protected file on a secure computer at Deakin University. Only the research team will have access to secured information. Only the de-identified data will be used in the analysis phase and in the summary of main outcomes that will be delivered to participants, any subsequent publications, and conference presentations. A data monitoring committee is not needed for this study as it is a non-therapeutic (behavioural) trial using low-risk procedures, and as such, the study team will be monitoring the data.

Analyses will be conducted using an intention-to-treat (ITT) approach [59], with all randomised participants analysed in their allocated group regardless of the intervention uptake. Every effort will be made to minimise missing data and where appropriate, multiple imputation will be used to handle missing data.

Baseline participant’s characteristics will be compared between the intervention and control group using Chi-squared or Fisher’s exact test for categorical variables, and *t* test or Kruskal-Wallis test for numerical variables. Drop-out bias will also be assessed using the same approach but comparing the baseline characteristics of participants with complete data against those lost to follow-up as a function of treatment group.

The intervention effect during the 12-week follow-up on numerical outcomes will be assessed using linear mixed models including group, time (T1, T2 and T3) and group by time interaction as fixed effects and participant as a random effect.

The feasibility of the Step One Program will be determined by the proportion of individuals who completed the baseline survey and were eligible to participate, how often participants read the SMS messages, how often they respond to Sunday SMS messages, and completion of follow-up surveys. Intervention acceptability will be reported by 1) summarising the multiple choice responses to the intervention acceptability questions in the T2 survey, and 2) performing qualitative analysis of the short answer responses and interviews using simple coding to identify recurring patterns and themes.

We will explore whether changes between T1 and T2 in the primary outcomes are correlated with changes in the potential mediator variables.

### Power analysis

The target sample size is 50 participants per group. Assuming 20% attrition rate, we estimate to collect complete data from 40 participants in each group. Sample size calculations are based on the only available Australian study of alcohol consumption in this population, the Australian Alcohol and lesbian/bisexual women – insights into culture and emotions (ALICE) study [1], which provides estimates for the AUDIT score. A sample size of 40 participants per group has 84% power for detecting a post-intervention mean change of 4 points in the AUDIT score, when the standard deviation is assumed as 6 for two independent groups, two-tailed test, and significance level 0.05. This sample size will achieve 80% power to detect effect sizes larger than 0.63 for any of the other scores outcomes.

## Discussion

SSAW tend to drink more than heterosexual women but are generally less likely to seek treatment [4, 24, 60, 61]. There are a range of barriers to SSAW accessing treatment [1], including a reluctance to attend mainstream clinical services, reports of low satisfaction with their care in these services, and difficulty finding services that are culturally tailored, sensitive and meet their needs [5–8]. Thus, an alcohol intervention that is culturally tailored and aware of issues specific to SSAW has the potential to increase access to support.

Few alcohol support services exist in Australia which are culturally tailored to SSAW or LGBT individuals. *Thorne Harbour Health* [62] run a therapeutic group for SSAW called *Drink Limits* and LGBT specific Alcoholics Anonymous (AA) meetings are available. However, limitations exist for many SSAW as these services are mainly urban based, and anonymity is reduced as the LGBT population is quite small. Thus, it is not surprising that in an Australian study exploring SSAW’s help-seeking preferences and behaviour (*n* = 1706), 55% of respondents reported that they use the internet for informal support [63]. SMS appears to be a viable option given research highlighting SMS as an effective method for delivering brief alcohol interventions in mainstream samples [13], as well as it overcoming many help-seeking barriers, including the option for anonymity, and broad access for women in both urban and rural locations.

The Step One Program was therefore developed using an Intervention Mapping framework to guide the process. While the co-design of this intervention is believed to increase the likelihood that SSAW will engage with it [64], we anticipate facing a number of operational issues during this study. Foremost is the potential difficulty recruiting the targeted number of participants given that minority groups can be difficult to reach [65, 66]. This is addressed by advertising the study in a variety of LGBT-specific and mainstream locations as described above under ‘Participant Recruitment’. Additionally, it is anticipated that recruiting participants for an alcohol intervention may also have its challenges as research has found SSAW avoid seeking alcohol reduction support as they fear stigma and judgement relating to their sexual orientation and problem with alcohol use [1, 6]. Therefore, the majority of advertising will be online via community and social networks through email, websites, and social media as they are discrete and do not require the individual to publicly take a flyer or write down the website. Furthermore, online advertisements present fewer barriers as the individual can open the survey straight away, whereas posters and flyers rely on an individual’s motivation to type the survey link into their phone or computer at a later time.

A final potential operational issue relates to the blinding of participants. That is, the study is being conducted in accordance with the CONSORT guidelines [67] and participants are blinded to the condition they are allocated to. Nonetheless, given that participants are aware that the study is trialling the effectiveness of a set of culturally tailored SMS messages, they are likely aware of which group they have been allocated to (i.e. the intervention or control group). This may bias the results on primary outcomes due to differential reporting in the intervention group. In addition, being in the control group, which involved completing the baseline survey and receiving a weekly SMS, may be an intervention in itself and act as a placebo effect. A similar outcome was reported from a trial of an online alcohol intervention developed in the United Kingdom for the general population [68]. The researchers were unable to demonstrate a significant difference between the control and intervention groups due to the fact that everyone reduced their alcohol intake [69].

Despite these potential operational issues, the significance of this research is underlined by insufficient knowledge among health practitioners regarding the specific needs and issues unique to SSAW despite their high risk for hazardous drinking and low satisfaction with care. Therefore, the results of this study may have important implications for clinical practice and provide direction for future research. This study will be the first to develop an empirically-based alcohol intervention specifically for SSAW and to provide evidence of their response to a brief alcohol intervention. Given the unique nature of this study, it is anticipated that the findings may inform policy makers of the feasibility and acceptability of a culturally tailored alcohol intervention for SSAW. Outcomes may also highlight the role of SMS interventions to facilitate alcohol reduction for SSAW, and promote resilience and wellbeing. Finally, this study may also inform and encourage the development of other health programs that are culturally tailored to SSAW, other specific groups within the LGBT community, or other marginalised hard to reach population groups.

## Abbreviations

AUDIT: Alcohol Use Disorders Identification Test
BRS: The Brief Resilience Scale
DASS: Depression Anxiety Stress Scales
LGBT: Lesbian, gay, bisexual, and transgender
MOS-SS: The Medical Outcomes Study Social Support Survey
PWI-A: The Personal Wellbeing Index – Adult
RCT: Randomised controlled trial
SMS: Short message service
SSAW: Same-sex attracted women
TNMS: The Treatment Needs and Motivation Scale
WHOQOL-BREF: The WHO Quality of Life-BREF
